# Deep learning-based morphological feature analysis and the prognostic association study in colon adenocarcinoma histopathological images

**DOI:** 10.3389/fonc.2023.1081529

**Published:** 2023-02-08

**Authors:** Xiao Xiao, Zuoheng Wang, Yan Kong, Hui Lu

**Affiliations:** ^1^ State Key Laboratory of Microbial Metabolism, Joint International Research Laboratory of Metabolic and Developmental Sciences, Department of Bioinformatics and Biostatistics, School of Life Sciences and Biotechnology, Shanghai Jiao Tong University, Shanghai, China; ^2^ Shanghai Jiao Tong University (SJTU)-Yale Joint Center for Biostatistics and Data Science, National Center for Translational Medicine, Shanghai Jiao Tong University, Shanghai, China; ^3^ Department of Biostatistics, Yale University, New Haven, CT, United States; ^4^ Center for Biomedical Informatics, Shanghai Children’s Hospital, Shanghai, China

**Keywords:** deep learning, colorectal cancer, prognostic prediction, morphological feature, gene ontology enrichment analysis

## Abstract

Colorectal cancer (CRC) is now the third most common malignancy to cause mortality worldwide, and its prognosis is of great importance. Recent CRC prognostic prediction studies mainly focused on biomarkers, radiometric images, and end-to-end deep learning methods, while only a few works paid attention to exploring the relationship between the quantitative morphological features of patients' tissue slides and their prognosis. However, existing few works in this area suffered from the drawback of choosing the cells randomly from the whole slides, which contain the non-tumor region that lakes information about prognosis. In addition, the existing works, which tried to demonstrate their biological interpretability using patients' transcriptome data, failed to show the biological meaning closely related to cancer. In this study, we proposed and evaluated a prognostic model using morphological features of cells in the tumor region. The features were first extracted by the software CellProfiler from the tumor region selected by Eff-Unet deep learning model. Features from different regions were then averaged for each patient as their representative, and the Lasso-Cox model was used to select the prognosis-related features. The prognostic prediction model was at last constructed using the selected prognosis-related features and was evaluated through KM estimate and cross-validation. In terms of biological meaning, Gene Ontology (GO) enrichment analysis of the expressed genes that correlated with the prognostically significant features was performed to show the biological interpretability of our model.With the help of tumor segmentation, our model achieved better statistical significance and better biological interpretability compared to the results without tumor segmentation. Statistically, the Kaplan Meier (KM) estimate of our model showed that the model using features in the tumor region has a higher C-index, a lower p-value, and a better performance on cross-validation than the model without tumor segmentation. In addition, revealing the pathway of the immune escape and the spread of the tumor, the model with tumor segmentation demonstrated a biological meaning much more related to cancer immunobiology than the model without tumor segmentation. Our prognostic prediction model using quantitive morphological features from tumor regions was almost as good as the TNM tumor staging system as they had a close C-index, and our model can be combined with the TNM tumor stage system to make a better prognostic prediction. And to the best of our knowledge, the biological mechanisms in our study were the most relevant to the immune mechanism of cancer compared to the previous studies.

## Introduction

Colorectal cancer(CRC) has become the third most commonly occurring cancer and a leading cause of cancer death worldwide (1). In the aspect of prognostic prediction, data-driven outcome prediction has demonstrated its promising power for making clinical decisions which could help doctors in providing treatment schedules and giving similar cases as references. Single modality data such as biomarkers and medical images have been proven effective in prognostic prediction for many studies (2–4). For example, in the field of medical images, Lewis et al. (3) proved that computer-extracted image features from surgical and biopsy tissue samples can help predict the level of disease aggressiveness in oropharyngeal squamous cell carcinoma. Grove et al. (4) quantitatively analyzed the computed tomography(CT) features from two independent cohorts, demonstrating that convexity score and entropy ratio were important prognostic factors for survival analysis, and were negatively correlated with overall survival in non-small cell lung cancer patients. Meanwhile, prognostic prediction based on biomarkers, such as transcriptome data and DNA methylation data, also plays an important role in helping physicians to identify patients with high mortality risk accurately (2, 5, 6). For example, by using the transcriptome data, Yi et al. (5) identified 7 key genes negatively correlated with the survival rate of rectal adenocarcinoma (READ). Yu et al. (6) found that DNA methylation level was negatively associated with the prognosis of bladder cancer patients and could be served as a potential biomarker. In recent studies, histopathological slide images especially the H&E stained slide had become a new research star. As the gold standard of diagnosis, histopathological slide images could provide rich information such as cell density, cell complexity, and immune infiltrations. Meanwhile, there exist more and more studies focusing on histopathological images and aiming to study their role in tumor prognosis prediction. With the help of computer-aided quantitative analysis of histopathological slide images, Cheng et al. (7) reported the association between specific image features and patient survival in gastric adenocarcinoma. In another research, Chen et al. (8) identified 12 features by Kaplan-Meier analysis that were significantly associated with 8-year disease-free survival.

Thanks to the advances in whole slide scanning technologies, we can access an unprecedented quantity of digital whole slide images with high resolution which could be up to 20x or 40x even. Along with a large amount of data from high-resolution whole slides, computer-aided whole slide analysis has become more and more noticeable. On the one hand, there may be some subtle features that are too hard to be recognized by human eyes which may contain essential information about diagnosis and clinical outcomes. Moreover, even an experienced pathologist can’t analyze tens of thousands of cells in one whole slide, nor quantitatively calculate the features of these cells. On the other hand, the computer vision processing algorithms together with the deep learning-based convolutional neural networks had greatly accelerated the computer-aided analysis of whole slide images from many aspects such as image classification (9), tumor region segmentation (10), tumor microenvironment analysis (11) and the end-to-end prognostic prediction (12), making the analysis process not only faster than manual work, but also more precise than the traditional way. However, due to the end-to-end characteristics of deep learning, studies may suffer from low interpretability (13), especially when the goal is not just to manipulate images (e.g. make prognostic predictions directly).

Morphological features of biological objects can be obtained by statistical calculation of characteristics of gray values such as geometrical characteristics and shape features. With the help of analysis tools such as CellProfiler (14), we can easily and quantitively obtain image features. The morphological features derived from the whole slide images provide another perspective of image data which displays in a matrix format, therefore, traditional machine learning can play a big role in mining the image features. For example, Chen et al. (15) used quantitative features of histopathological images for survival prediction of clear cell renal cell carcinoma. Yin et al. (16) used a machine learning approach to analyze features extracted by CellProfiler from whole slides and achieved an accuracy of 91%-96% in distinguishing tumor stage Ta and T1. However, due to the limitation of computing power and the software itself, previous studies only used small pieces from whole slide images regardless of whether the pieces belonged to the tumor region or the non-tumor region. Considering that most prognosis studies of cancer mainly focus on the tumor region (17), the averaged morphological features from the confused small pieces of tumor and stromal region possibly lead to less trustworthy results or less accurate prediction results, which may have a negative influence on the statistical significance and the biological interpretability of the model. To avoid the potential risks, selecting small pieces from the same type of region may serve as an effective way. In addition, although there existed some studies exploring the biological significance and the interpretability of the prognosis prediction model based on quantitative morphological features, the results were not closely related to the mechanisms of cancer immunology (18).

In our study, we built a prognostic prediction model using morphological features from the H&E stained slides. We also proved that our prognostic prediction model with deep learning-based tumor segmentation had better statistical significance and better biological interpretability compared to the model without tumor segmentation.

## Material and methods

### Data sets and data preprocessing

**Figure 1:**
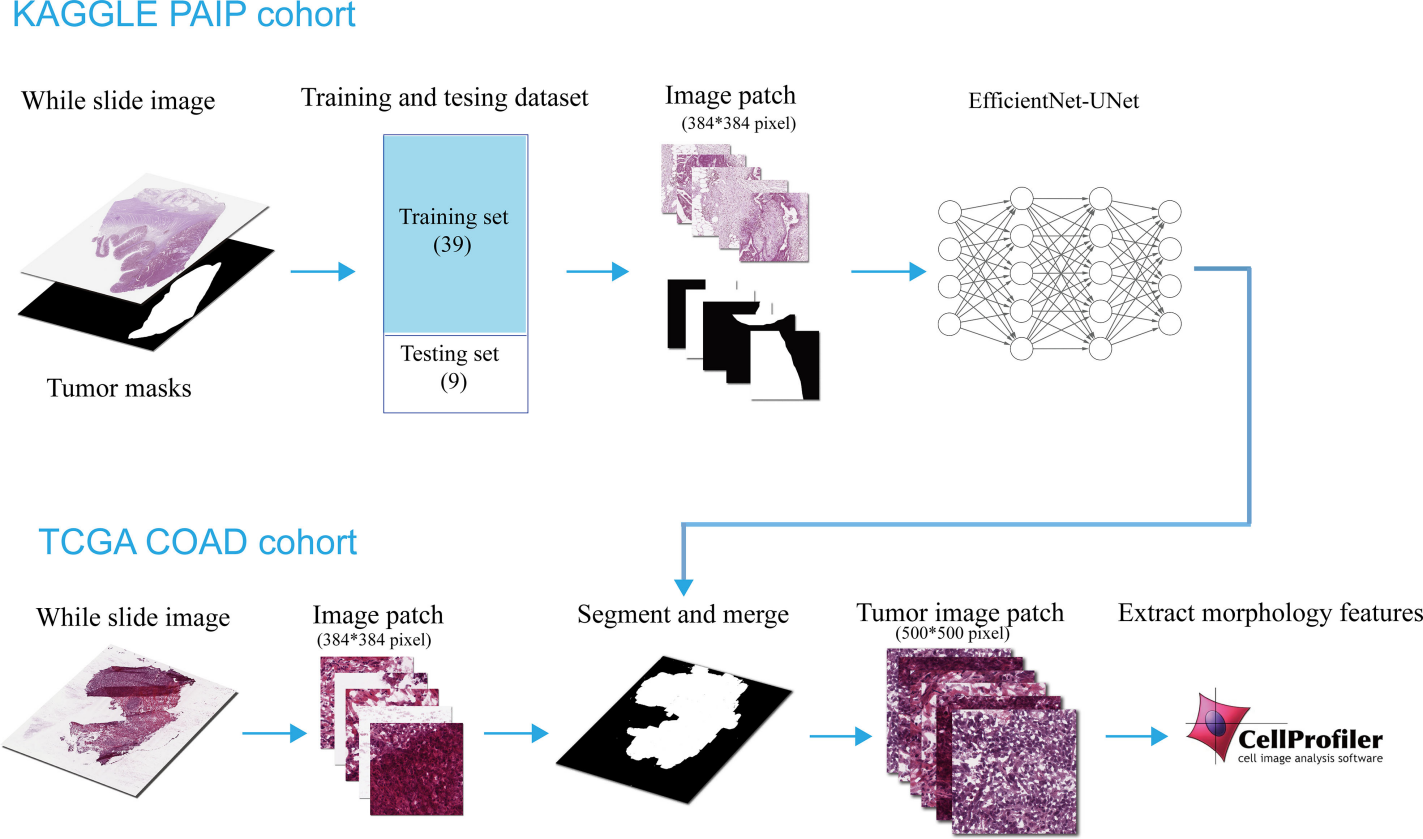
Flowchart of the construction and use of the tumor region segmentation deep learning model.

We acquired hematoxylin and eosin (H&E)-stained histological slide images for patients with colon adenocarcinoma (COAD) from The Cancer Genome Atlas (TCGA) data portal as the object of our analysis. We downloaded all available 1560 slides with svs formats. Patients’ transcriptome data and their clinical records (**Table 1**) were also used for analysis. The whole analysis process was shown in **Figure 1**.

**Table 1:** The clinical information of the TCGA-COAD cohort (selected 385 patients).

Characteristics	
**Male/female, n**	203/182
Age, years	
30-49	45 (12%)
50-69	167 (43%)
>70	173 (45%)
**Alive/dead, n**	297/88
Pathologic stage, n	
Stage I	65 (17%)
Stage IA	1 (0.3%)
Stage II	22 (5.7%)
Stage IIA	117 (30%)
Stage IIB	9 (2.3%)
Stage IIC	1 (0.3%)
Stage III	17 (4.4%)
Stage IIIA	7 (1.8%)
Stage IIIB	51 (13%)
Stage IIIC	36 (9.4%)
Stage IV	35 (9.1%)
Stage IVA	15 (3.9%)
Stage IVB	2 (0.5%)
Unknown	7 (1.8%)
Depth of tumor (T stage), n	
T1	9 (2.3%)
T2	67 (17%)
T3	262 (68%)
T4	22 (5.7%)
T4a	16 (4.2%)
T4b	8 (2.1%)
Tis	1 (0.3%)
N stage, n	
N0	228 (59%)
N1	64 (17%)
N1a	11 (2.9%)
N1b	15 (3.9%)
N1c	2 (0.5%)
N2	47 (12%)
N2a	6 (1.6%)
N2b	12 (3.1%)
M stage, n	
M0	288 (75%)
M1	40 (10%)
M1a	9 (2.3%)
M1b	3 (0.8%)
MX	40 (10%)
Unknown	5 (1.3%)
Race, n	
American indian or alaska native	1 (0.3%)
Black or african american	55 (14.3%)
White	210 (54.5%)
Asian	11 (2.9%)
Not reported	158 (41.0%)
Ethnicity, n	
Hispanic or latino	4 (1.0%)
Not hispanic or latino	248 (64.4%)
Not reported	133 (34.5%)

For the tissue histological slide images, we performed a quality control step and excluded the slide with low quality. The whole slide image data in the TCGA-COAD dataset was stored in the format of svs, where the images were compressed with different resolutions at different levels. And a low number of levels and low resolution at level 1 would indicate poor quality of the corresponding whole slide image. So we first excluded slides with less than 2 compress levels, then removed the slides with less than 5000 x 5000 pixels size at level 1. After that, we got 941 slides for 460 patients, of which 459 patients have the transcriptomic data. Then, the patients whose overall survival information was lost were excluded (e.g. a patient whose vital state is “alive” but whose days to last follow-up was 0).

### Tumor region segmentation model

#### Training and testing data

In this part, we used the dataset from the Pathology AI Platform (PAIP) 2020 challenge as the training and validation data for our tumor region segmentation model. In this dataset, all patients were histologically diagnosed with COAD, and all cases including colon tumor tissues were diagnosed at SNUH, SNUBH, and SMG-SNU BMC between January 2005 and June 2018. In terms of images, there were 47 whole slide images (WSIs), and all of them were obtained by hematoxylin-eosin staining (H&E) and scanned at 40X magnification using the Aperio AT2. The labels of tumor regions, which were defined as boundary enclosing dispersed visible tumor cell nests, necrosis, and peri- and intratumoral stromal tissue, were marked by experienced pathologists and stored in the format of XML.

We then divided the 47 WSIs into training data with 39 WSIs and validation data with 8 WSIs. The raw and masked WSIs in both the training and testing set were split into small patches with 384*384 pixels. Before feeding into the segmentation model, all small patches were filtered to minimize the number of images from the background region.

#### Neural network architecture

**Figure 2:**
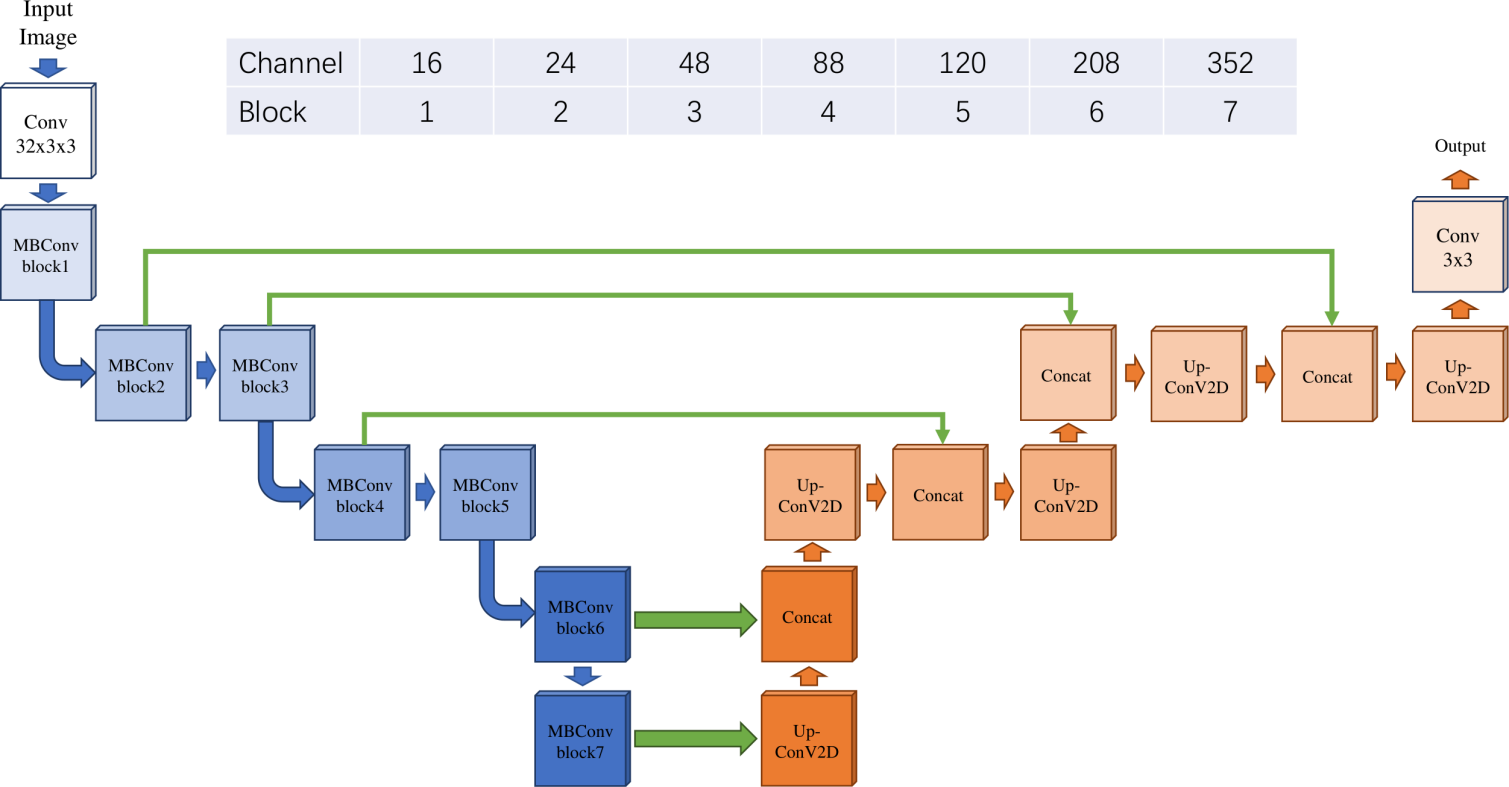
The architecture of Eff-Unet with EfficientNet B2 as the encoder.

In this study, Eff-Unet (19) (version B2) was used to perform the tumor segmentation, which achieved the benchmark performance and won the first prize in the IDD Lite segmentation challenge. The architecture of the Eff-Unet was derived from the famous U-Net (20), which is a symmetric U-shaped fully convolutional neural network developed for biomedical image segmentation. Instead of using a traditional set of convolution layers, the Eff-UNet employs EfficientNet (21) as the encoder, feature extractor, and decoder of UNet, which combines the high-level features and the low-level spatial information for more exact pixel-wise segmentation. The detailed architecture of Eff-Unet is shown below in **Figure 2**.

For the training step, we used the Adam optimizer for 200 epochs with a batch size of 18 and fixed learning rate of 0.001 on a single GPU, and a weight decay of 1e-4, which was the same as the hyperparameters mentioned in the Eff-Unet article (19). Moreover, the BCEDiceLoss was chosen to be the loss function, which was the combination of the loss function of DICE loss and BCE loss and has had a good performance in semantic segmentation tasks (22). All training was conducted on NVIDIA V100 GPU with 16GB memory on Linux CentOS 7.

### Tumor region prediction

The slides from the TCGA-COAD cohort were first loaded and preprocessed as described in 2.2.1, they were then cropped into thousands of small tiles with 384x384 pixel size on the level 1 scale. Next, these small pieces were fed into the tumor region segmentation model to predict the tumor masks. And finally, the predicted results of small tiles were combined into a complete tumor region-predicted mask.

We enlarged the predicted mask from the level 1 scale to the same dimension as the slide on level 0. And then 30 small tiles with a size of 500 x 500 pixels were selected randomly from the predicted tumor region. In addition, for the patients who have multiple tissue slides, the 30 tumor region was randomly selected from all slides. These 30 tiles would serve as a representation of one patient.

In addition, following the same methods above, we also randomly selected 30 tiles from patients’ whole slides to serve as their representation. Notably, background regions with no cells were excluded from all the selected regions.

### Morphological features

Following the process steps proposed by Luo et al. (23), we calculated the morphological image features using CellPorfiler, which is considered a state-of-the-art medical image analysis tool. The extracted morphological features included cell size, shape, and texture of the nuclei, as well as the distribution of pixel intensity in the cytoplasm and nuclei. These features included some standard features of histopathological image analysis (24) and complex features like Zernike shape features, and Haralick and Gabor texture features, such as “AngularSecondMoment” and “Texture_Entropy”, which have been proven helpful to characterize the tumor habitats (25, 26). Additionally, we also extracted features that measure an image as a whole in some aspects, such as intensity, texture, saturation, blur, and the area occupied by the stain. Then, CellProfiler will automatically calculate the statistical characteristic (e.g. mean value, median, standard deviation) of the morphological features of each cell in an input picture. After that, we performed the patient-wise average calculation to get the patient-level morphological features from the selected 30 patches. And finally, the values of these morphological features were transformed with MinMax normalization to better adapt to the prognostic analysis.

#### Survival analysis for different models

We performed survival analysis to assess the prognostic value of morphological features. The Lasso-Cox model, which can automatically adapt to the negative influence of co-linearity, was used to select prognosis-related features. Notably, the features we selected were the set of features that had the lowest partial likelihood deviance in cross-validation among different random seeds. Then, features with the same measuring method but with a different computing method by CellProfiler were removed to eliminate feature redundancy in the model and further eliminate co-linearity to the prognostic prediction model (e.g. StDev_Identifyhemasub2_Texture_AngularSecondMoment_ImageAfterMath_3_01_256 and StDev_Identifyhemasub2_Texture_DifferenceEntropy_ImageAfterMath_3_02_256). In addition, the BIC criterion was used to optimize variable selection.

We performed a cox regression and used the KM survival curve to evaluate the effectiveness of our model in the TCGA-COAD cohort. We first fit a multivariate cox regression model with the prognosis-related features selected above. The prognostically significant features were those with a p-value less than 0.05 in the multivariate cox regression The risk score of each patient was defined as the weighted sum of the patient’s selected features, weighted by the coefficients in the multivariate cox model (15). Patients were then divided into high- and low-risk groups with a cutoff value at the median of all patients’ risk scores. KM survival curve was assessed in both the high- and low-risk groups. The overall survival difference between the two groups was evaluated using the log-rank test.

Next, we performed cross-validation to better evaluate the effectiveness of the prognostic model. Patients in the TCGA-COAD dataset were divided into training and testing sets of the same size. Given that there were 22% of patients with a death event, stratified randomization was performed in order to balance the proportion of death in each set. We then trained a cox regression model using patients’ survival and risk scores in the training set. Then, we calculate the risk scores of patients in the testing set using the weights obtained from the cox regression model trained by the training set. Finally, the KM survival curves and the overall survival difference were calculated, and the cross-validation was performed 50 times with different random seeds.

Using the feature selection procedures described above, we evaluated the correlation between patients’ prognosis and their morphological features extracted from cells in the tumor region and cells randomly selected from the whole slide. We also compared these two models using KM survival curves and cross-validation. In addition, we further compared our model with the prognostic prediction model using clinical features and that using the TNM tumor staging system suggested by the American Joint Committee on Cancer (AJCC) and the Union for International Cancer Control (UICC). In these statistical analyses, the Lasso-Cox model was conducted using the R package glmnet (27) and all analysis was performed using R 4.1.3.

#### Functional enrichment analysis

To evaluate the biological significance of our prognostic prediction model, we performed functional enrichment analysis to investigate whether the genes correlated with those prognostically significant morphological features are enriched in various biological mechanisms. We first calculated spearman correlations between the expression levels of all genes and patients’ morphological features. Genes were considered significantly correlated with a morphological feature if the FDR-adjusted p-value of the spearman correlation was less than 0.01. Then, Gene Ontology (GO) enrichment analysis was performed to identify the related biological mechanisms of those significant genes using the R package clusterProfiler (28) on the organism “Homo sapiens”. Lastly, we compared the list of biological mechanisms revealed by the prognostically significant features selected from the model with tumor segmentation and without tumor segmentation.

## Results

### Tumor region segmentation and morphological features extraction

We evaluated the tumor region segmentation model on training and validation set using the intersection over union (IOU) index. The average IOU value of 0.866 on the training set and 0.833 on the validation set implied the validity of our model. In addition, the minimum value of IOU on the training set was 0.710 and 0.739 on the validation set, which also proved the accuracy of our segmentation model.

As an independent image dataset, 941 slides from patients in the TCGA-COAD cohort were processed and segmented for the predicted tumor region (**Figure 1**).

After the processing and filtering steps, the morphological features of small patches from the tumor region and regions randomly selected from the whole slide were then extracted. In total, we extracted 571 morphological features including tissue textures, nuclei, cells, cytoplasm, and the neighboring architecture. The feature matrixes for the tumor region and randomly selected regions will be shown separately in **Supplementary Table 1** (tumor region), and **Supplementary Table 2** (randomly selected regions).

We at last retained 385 patients who have the corresponding qualified slides, transcriptomic data, and survival information. Of all the patients, the race, ethnicity, age, sex, vital status, and cancer stage of patients in the TCGA-COAD cohort were summarized in **Table 1**.

### Evaluation of different prognosis models

**Figure 3:**
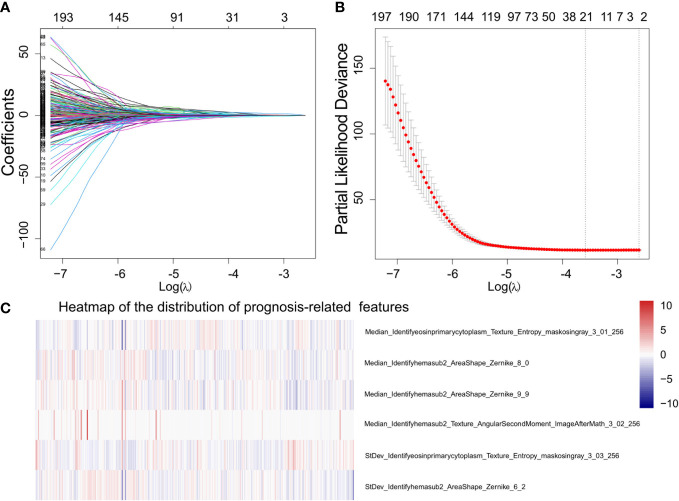
Demonstration of the prognosis-related morphological feature selection process using the Lasso-Cox model. **(A)** The profile of coefficients in the model at different levels of L1 penalization plotted against the log(lambda) sequence. **(B)** The minimum standard (the left line) was adopted to obtain the value of the super parameter λ by 10-fold cross-validation with the criteria of partial likelihood deviance. **(C)** Heat map that shows the distribution of prognostically significant features.

**Figure 4:**
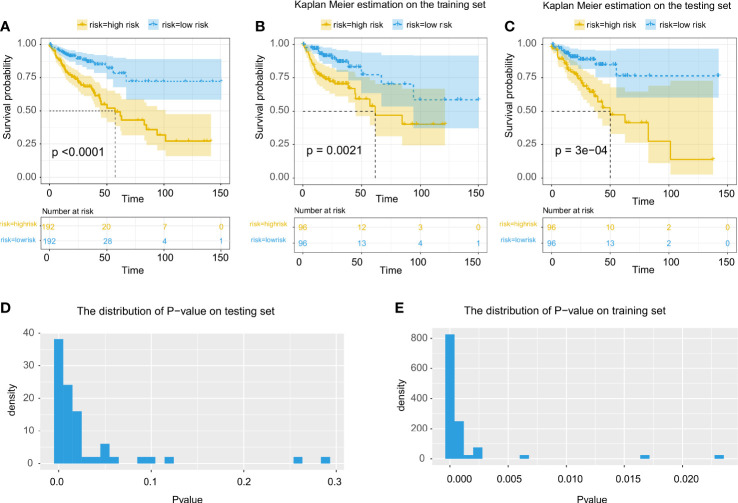
Evaluation of the prognostic prediction model using features extracted from cells in tumor region. **(A)** Multivariate cox regression for all patients using all prognosis-related morphological features. **(B, C)** Kaplan Meier estimate using multivariate cox regression on training and testing data. **(D, E)** Statistical evaluation and demonstration of the prognostic prediction model using selected prognosis-related morphological features on the testing set and the training set.

Using the tumor region segmentation model, the number of prognosis-related features selected by the Lasso-Cox model was increased from 5 to 12. We further excluded 1 feature out of the 12 features because 2 features were measured by the same method by CellProfiler (**Supplementary Table 3**). Among the selected 11 features, the multivariate cox regression model produced 5 morphological features that were prognostically significant for the model with tumor segmentation (**Table 2**; **Figure 3**), whereas there were only 3 prognostically significant features in the model without tumor segmentation. The KM survival and its C-index demonstrated significant overall survival difference between the high- and low-risk groups in the model with tumor segmentation (p-value = 1.5e-6, C-index = 0.701, **Figure 4A**), which is more statistically significant compared to that of the model without tumor segmentation (p-value = 6.4e-05, C-index=0.663). It is also worth mentioning that the normalization method we used was the 0-1 normalization, so there might be some outliers that made the other values relatively small, and that may cause the range of HR estimates different from each other.

**Table 2:** The key information of the prognostically significant morphological features in the multivariate cox regression.

Feature	HR	HR.95L	HR.95H	P-value
Median_Identifyeosinprimarycytoplasm_Texture_Entropy_maskosingray_3_01_256	289.6413	10.84684	7734.237	0.000719
Median_Identifyhemasub2_AreaShape_Zernike_8_0	0.141232	0.025642	0.777883	0.024544
Median_Identifyhemasub2_AreaShape_Zernike_9_9	102.0159	7.546394	1379.101	0.000499
Median_Identifyhemasub2_Texture_AngularSecondMoment_ImageAfterMath_3_02_256	16.6161	1.134321	243.4007	0.040171
StDev_Identifyeosinprimarycytoplasm_Texture_Entropy_maskosingray_3_03_256	13.99343	1.598739	122.4817	0.017131
StDev_Identifyhemasub2_AreaShape_Zernike_6_2	86.34095	3.582171	2081.073	0.006036

Through cross-validation (**Figures 4B, C**), the prognostic model with tumor segmentation performed better than the model without tumor segmentation (**Figures 4D, E**). For the KM estimate, we observed a significant overall survival difference (p-value < 0.05) in all the testing sets for the model with tumor segmentation, whereas only 92% of the testing sets showed a significant overall survival difference for the model without tumor segmentation.

The comparison of the C-index, the p-value of the KM estimate, the number of prognosis-related features and prognostically significant features, and the results of cross-validation were summarized in **Table 3**.

**Table 3:** Comparison of C-index of the cox regression model, -log10(p-value) of survival analysis, the number of prognosis-related features, and the percentage of p-value less than 0.05 on the training and testing set in cross-validation between the prognostic prediction model using morphological features extracted from cells in the tumor region and the model using features in regions randomly selected from the whole slide.

Region	C-index of cox regression	-log10(P-value of KM estimate)	Number of prognosis-related features	Number of prognostically significant features	Percentage of P-value less than 0.05 on the training set	Average p-value on the testing set
Tumor	0.7007	5.825	11	6	100%	3%
Randomly selected	0.6637	4.191	5	3	92%	7%

In addition, we found that our prognostic prediction model was almost as good as the TNM tumor staging system as they had a close C-index. We also found that when combining the morphological features in the tumor region with the TNM stage clinical features, the prognostic model using both features outperformed the model that only used TNM stage clinical features (**Table 4**). Considering that it would take experienced doctors some time to make decisions on the TNM stage but the computer can automatically perform the procedures mentioned above, our model using morphological features in the tumor region is expected to be helpful in real clinical practices.

**Table 4:** Comparison of the prognostic prediction models using morphological features, age and gender, and the TNM tumor stage.

Model	C-index
Age(year)+Gender	0.579
Ajcc tumor stage	0.739
Morphological feature	0.701
Morphological features and Ajcc tumor stage	0.757

### Enrichment analysis

**Figure 5:**
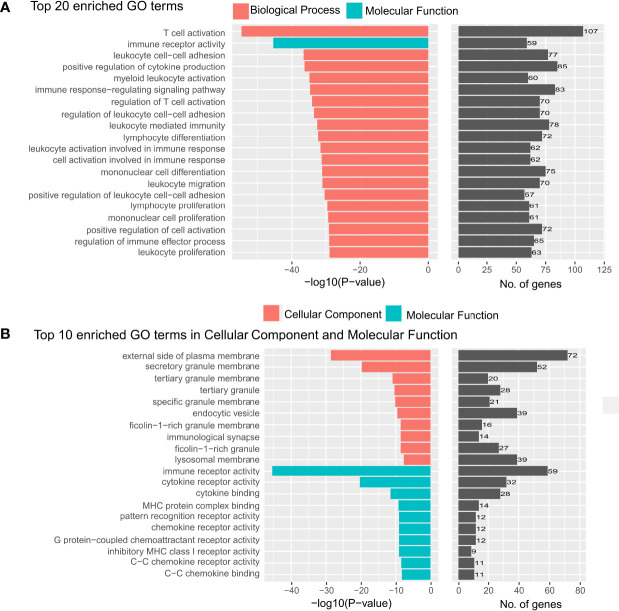
Top 20 gene ontology terms in the enrichment analysis. **(A)** The top 20 gene ontology (GO) terms that were significantly enriched in the enrichment analysis on genes that are related to the prognostically significant features in the model with tumor segmentation. **(B)** The top 20 GO terms which were significantly enriched in “cellular component” and “molecular function”.

For the prognostically significant features extracted from cells in tumor regions, there were a total of 645 unique genes that were correlated with one or more morphological features (**Supplementary Table 4**). GO enrichment analysis revealed that these correlated genes were mainly enriched in “T cell activation and regulation”, “leukocyte cell-cell adhesion”, “leukocyte proliferation and migration” and “immune receptor activity” (**Figure 5A**), indicating the abnormal pathway in immune escape and tumor spread. In addition, we found that except for one GO term belonging to “molecular function”, all the other top 20 enriched GO terms belong to “Biological Process” (BP). The top 10 enriched GO terms in “Molecular Function” (MF) and “Cellular Component” (CC) were also analyzed (**Figure 5B**). All of them are closely related to the immune mechanisms of cancer, and corresponded to the pathway of immune escape and tumor spread. To the best of our knowledge, the biological significance revealed by our study was the most related to the immune mechanisms of cancer compared to previous studies (18, 29) which used morphological features to make the prognostic prediction of cancer patients.

**Figure 6:**
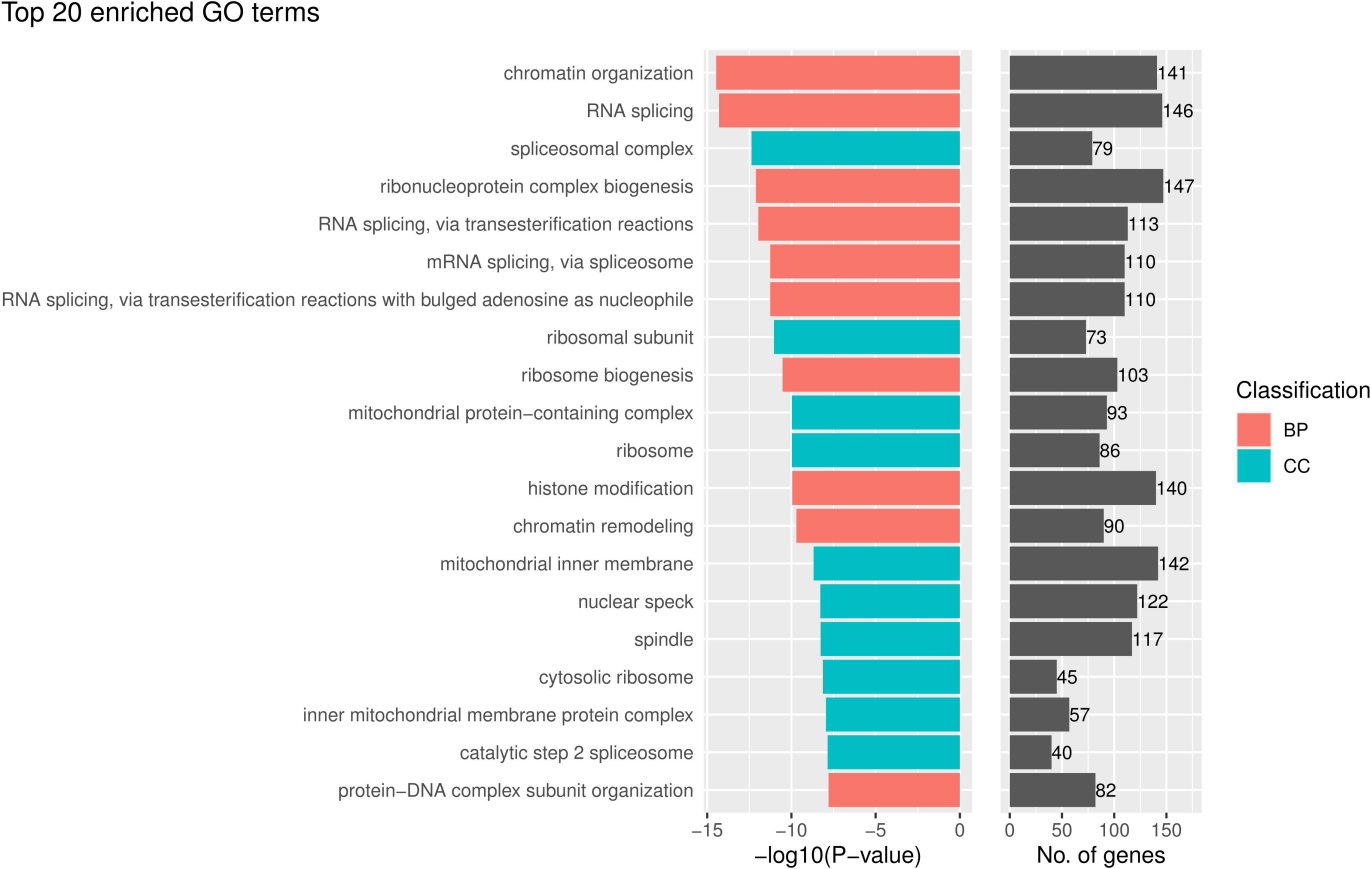
The top 20 gene ontology (GO) terms that were significantly enriched in the enrichment analysis for genes that are related to the prognostically significant features in the model without tumor segmentation.

In contrast, without tumor segmentation, the model using features from pieces randomly selected in the whole image does not have a very interpretable biological meaning. The pathways in the GO enrichment analysis on the genes correlated with the selected features in the model without tumor segmentation mainly were GO terms relevant to RNA splicing and ribosome (**Figure 6**) but not directly related to cancer immunology. And the p-values of these GO terms were much larger than that of the model with tumor segmentation.

## Discussion

In this study, we did a prognostic prediction study for colorectal cancer using morphological features of cells in the H&E-stained slides. With a higher C-index, a much lower p-value, and better performance on cross-validation, our prognostic prediction model with tumor segmentation has a better statistical significance than the model without tumor segmentation. We also looked at the association between the expressed genes and the prognostically significant morphological features. And we found that those correlated genes were enriched in the aspects of “leukocyte cell-cell adhesion”, “leukocyte proliferation and migration” and “immune receptor activity”, which is more relevant to the immune mechanisms of cancer compared to that of the model without tumor segmentation. In addition, these pathways were also the most related to cancer immunology compared to previous studies, which demonstrated good biological interpretability of the model with tumor segmentation. This may indicate how the genotype may affect the phenotype and how those genes may affect the prognostic.

The image process step in our study was accurate and the image type we use was interpretable. With the development of more accurate and more interpretable AI algorithms, deep learning has shown its magnificent power in dealing with biomedical images, such as tumor region identification, metastasis detection, and patient prognosis (30). Also, pathological images may be more insightful, interpretable, and sensitive than radiometric images because alterations in tumor molecular pathways are frequently mirrored in cell shape (18). Here, for our tumor region segmentation part, we utilized the Eff-UNet as our tumor region segmentation model for the H&E stained pathological images. By applying the state-of-art Efficientnet B2 as the encoder and decoder of U-net and using the BCEDiceLoss as our loss function, we achieved the average IOU of 0.87 and 0.83 on the training and validation set, which indicated a good performance of our tumor region segmentation model compared to traditional models (31).

The prognostic prediction model using features from the tumor region other than randomly selected from the whole slide was reasonable and has been proven effective. There are tumor regions and non-tumor regions in the WSI, and the morphological features of cells in the normal tissue regions vary so far from the tumor region that experienced doctors can manually distinguish the tumor region from the non-tumor region. As a result, the prognosis prediction will be compromised when using the morphological features from confused tissues both from tumor regions and non-tumor regions. Theoretically, as the mutations in cancer cells, changes in tumor molecular processes, and other decisive factors for the diagnosis and prognosis of cancer patients are always mirrored in the tumor region instead of everywhere in the whole slide, it is reasonable that the morphological features of cells in the tumor region would contribute more to the prognosis of patients. In practice, we proved that the model using features from tumor regions was more statistically significant than the model using features from other regions. Also, the biological significance revealed by the model with tumor segmentation, which was more related to the immune mechanisms of cancer compared to that of the model without tumor segmentation, proved the improvement brought by tumor segmentation. To sum up, by using the deep learning tumor segmentation model to distinguish the tumor region from the non-tumor tissue, we overcame the drawback of previous work where the features were extracted from cells randomly cropped from the whole slide, making our prognostic prediction model more reasonable, explainable and accurate.

The statistical method used in our study was effective and the prognostic prediction model in our study was very statistically interpretable. In the morphological feature selection part, we used the Lasso-Cox model to identify the prognosis-related features, which reduced the damage of co-linearity to the model and comprehensively selected the truly significant features. We also excluded features with the same measuring method but with a different computing method by CellProfiler, which reduced the redundancy of the selected morphological features and further solved the problem of co-linearity. After the selection analysis of significant morphological features, we discovered 6 features that would significantly make the prognosis worse or better. Divided by the object that the features describe, 4 of these features were from nucleus, including the features of texture and area shape, and 2 were features of cytoplasm. When divided by the statistical meaning of these features, 2 of these features are the standard deviation of cells’ morphological features, which may indicate how much the cells in the tumor region vary from each other, and may further indicate the tumor’s microenvironment. And 4 of these features were the median value of cells’ morphological features, which refer to the average texture feature of the tumor, and may further indicate how the overall texture of the cells in the tumor region affects the prognosis. Also, as it is hard for pathologists to estimate or quantitatively calculate these morphological features, this accurate prognosis prediction model may provide a new way for pathologists to make the prognostic prediction.

The prognostic prediction model in our study also revealed some biological mechanisms closely related to the immune mechanisms of cancer, such as immune escape and the spread of the tumor, which indicates better interpretability. After the identification of the prognostically significant morphological features, we further explored the biological mechanisms hidden in those morphological values. In the Gene Ontology analysis, the “Biological Process” was the dominant part among “Molecular Function”, “Biological Process” and “Cellular Component”, with a p-value much less than other parts. The results of enrichment analysis mainly focus on the activation and regulation of T-cells and leukocytes, proliferation of immune cells, regulation of cell-cell adhesion, and immune receptor activity. These all lead to a worse prognosis for the following reasons. On the one hand, it is clear that the abnormal regulation of T-cells and leukocytes will cause tumor immune escape (32), which leads to a worse prognosis. While on the other hand, recent studies have proven that the abnormal regulation of immune cells, together with the proliferation of immune cells, will instead promote the growth of cancer cells. To be more precise, chronic inflammation is caused by persistent immune system activation and the failure of the inflammatory response to resolve. The chronic inflammatory microenvironment then promotes tumor growth and genomic lesions which also leads to a worse prognosis (33). Also, the changes in cell-cell adhesion would make the cancer cells leave their original position and spread to other organs, which is a marker of the formation of malignant tumors (34). To sum up, all of these biological processes have been proven crucial in the development and progression of cancer cells, which shows the interpretability of our prognostic model based on morphological features.

The biological mechanisms of the molecular function part and the cellular component part can also give us insight into the biological mechanisms of cancer, where they also indicate the mechanisms of immune escape and the spread of the tumor. In terms of cellular components, the enriched GO terms mainly focus on the granule membrane, plasma membrane, and immunology synapse. These all have a relation to the biological membrane and may be reflected in the plasma morphological features of cells in the tumor region. As for the granule, when cells are exposed to stress stimuli, stress granules (SGs) are one organelle that cells will form to aid cells in coping with stress. And it has been proven that SGs function not only to regulate the switch between survival and cell death, but also contribute to cancer cell proliferation, invasion, metastasis, and drug resistance (35). There has also been evidence suggesting that the expression and/or activity of several key SGs components is deregulated not only in colorectal tumors but also in pre-neoplastic conditions (e.g., inflammatory bowel disease), implying a potential role in the development of CRC (36). Also, the GO term “external side of plasma membrane”, containing any biomolecule embedded or anchored in it or attached to its surface, plays an important role in the immune mechanisms of cancer. Studies have proven the abnormal external side of the plasma membrane can lead to a worse prognosis for patients with cancer in many ways: higher cell proliferation, disturbances in signaling pathways, and prevention of the cells from demanding conditions of the microenvironment (37). As for GO terms “immunology synapse”, it has been proven that the host would be vulnerable to pathogens or tumor escape at one extreme and suffer from autoimmunity at the other extreme if the immunology synapse is dysregulated (38), which also makes immunology synapse crucial for the prognosis of patients with cancer.

In terms of molecular function, immune receptor activity, C-C chemokine binding/receptor activity, cytokine binding/receptor activity, and MHC protein complex binding/receptor activity are the top aspects of the enriched GO terms, where all of them also lead to a better or worse prognosis and are closely related to the immune mechanisms of cancer. The malfunction of the immune receptor and immune recognition receptor activity will also lead to immune escape and worsen the condition and so does the mutation of C-C chemokine. There has been evidence indicating that the expression of C-C chemokine receptor 7 in malignant tumors promoted migration to the lymph nodes (39), which will make the prognosis worse. And for the GO term “cytokine binding”, because some cytokines bind to particular G-protein-coupled seven-span transmembrane receptors, which are important regulators of cell trafficking and adhesion, abnormal cytokine binding/receptor activity, cytokines’ malfunction may promote the spread of cancer cells and worsen prognosis (40) thus leading to a worse prognosis. This mechanism also corresponds to the GO term “G protein−coupled chemoattractant receptor activity” in the MF part. And when it comes to the MHC protein complex binding/receptor activity, they are in charge of binding peptides derived from a cell’s expressed genes, then transporting, and displaying this antigenic information on the cell surface, so that CD8 T cells can recognize pathological cells that are synthesizing abnormal proteins, such as cancers that are expressing mutated proteins (41). As a result, losing MHC I antigen presentation machinery is one way where cancers can evade immune control, which also has a negative impact on prognosis.

There are also some limitations in this study. First of all, we only use the data from TCGA-COAD, where the amount of data is limited and more data would make the research more convincing. Also, some patients do not have the whole slide image that contains the non-tumor region, thus the evaluation of the model using features from only the non-tumor region was unable to carry out. Secondly, we used the method of treating the median risk score as the cut-off value to divide the patients into the high-risk group and the low-risk group, where a specific value is not required.

## Conclusions

In conclusion, we developed and verified a robust prognosis prediction model using the morphological features of patients’ H&E-stained pathological images. We proved that with the help of our tumor region segmentation deep learning model, using the features extracted from cells in the tumor region will make a better prognostic prediction than using features of regions randomly selected from the whole slides. With a good performance on survival analysis and cross-validation, our model with tumor segmentation can very accurately make the prognostic prediction. The biological interpretability is also proven to be better by segmenting the tumor region. As the results of enrichment analysis for genes that are related to the tumor region’s prognostically significant features highly correlated with cancer immunology, our study demonstrated good biological interpretability of our model and may provide insight into the relationship between cancer’s phenotype and the biological mechanisms of cancer.

## Data availability statement

The original contributions presented in the study are included in the article/**Supplementary Material**. Further inquiries can be directed to the corresponding authors. The datasets analyzed for this study can be found in the TCGA-COAD.

## Author contributions

XX participated in the data acquisition, performed the statistical analysis, and drafted the manuscript. YK conceived of the study and participated in its design. ZW supervised the biostatistics analysis, and all aspects of the study were supervised by HL and YK. All authors contributed to the article and approved the submitted version.

## Supplementary material

Supplementary File 1 The parameters we used for feature extraction for the software CellProfiler.

Supplementary Table 1 The morphological features extracted by CellProfiler from cells in the tumor region.

Supplementary Table 2 The morphological features extracted by CellProfiler from cells in regions randomly selected from the whole slide.

Supplementary Table 3 The morphological features associated with prognosis.

Supplementary Table 4 The correlation between patients’ prognostically significant morphological features and their expressed genes.
